# Demographic, seasonal, and spatial differences in acute myocardial infarction admissions to hospital in Melbourne Australia

**DOI:** 10.1186/1476-072X-7-42

**Published:** 2008-07-30

**Authors:** Margaret E Loughnan, Neville Nicholls, Nigel J Tapper

**Affiliations:** 1 School of Geography and Environmental Science Monash University, Wellington Road Clayton, Australia

## Abstract

**Background:**

Seasonal patterns in cardiac disease in the northern hemisphere are well described in the literature. More recently age and gender differences in cardiac mortality and to a lesser extent morbidity have been presented. To date spatial differences between the seasonal patterns of cardiac disease has not been presented. Literature relating to seasonal patterns in cardiac disease in the southern hemisphere and in Australia in particular is scarce. The aim of this paper is to describe the seasonal, age, gender, and spatial patterns of cardiac disease in Melbourne Australia by using acute myocardial infarction admissions to hospital as a marker of cardiac disease.

**Results:**

There were 33,165 Acute Myocardial Infarction (AMI) admissions over 2186 consecutive days. There is a seasonal pattern in AMI admissions with increased rates during the colder months. The peak month is July. The admissions rate is greater for males than for females, although this difference decreases with advancing age. The maximal AMI season for males extends from April to November. The difference between months of peak and minimum admissions was 33.7%. Increased female AMI admissions occur from May to November, with a variation between peak and minimum of 23.1%. Maps of seasonal AMI admissions demonstrate spatial differences. Analysis using Global and Local Moran's I showed increased spatial clustering during the warmer months. The Bivariate Moran's I statistic indicated a weaker relationship between AMI and age during the warmer months.

**Conclusion:**

There are two distinct seasons with increased admissions during the colder part of the year. Males present a stronger seasonal pattern than females. There are spatial differences in AMI admissions throughout the year that cannot be explained by the age structure of the population. The seasonal difference in AMI admissions warrants further investigation. This includes detailing the prevalence of cardiac disease in the community and examining issues of social and environmental justice.

## Background

Several studies have described a seasonal fluctuation in cardiac mortality and morbidity in the northern hemisphere with increased rates during the colder months [1-5]. Winter increases in Acute Myocardial Infarction (AMI) mortality in America were first described in 1937 [6,7]. Subsequent studies from Europe, America, and Asia have supported these observations [2,4,8-13]. Recently, studies have examined the differences in age and gender distributions in AMI mortality and morbidity [14-17]. These studies suggest that the influence of age on seasonal variation in AMI mortality and morbidity becomes important from age 55 years onwards. Also, being male increases the risk of AMI, although this effect is known to decrease with advancing age. Additionally, over the last decade hospital admissions for coronary heart disease have been influenced by changes in diagnostic technology that has lowered the threshold for defining AMI events [18]. Although this may contribute to overall numbers of admissions it is unlikely to be seasonally related.

Whilst descriptions of seasonal variation in AMI mortality and morbidity are not new, there is little information available detailing the existence or pattern of seasonal trends in Australia and none relating to south-eastern Australia where climate variability is most pronounced [19-22]. We therefore are still unsure if there are temporal, demographic, and spatial differences within AMI morbidity in Melbourne, i.e. whether or not persons suffering AMI in either warmer or colder months are drawn from the same population. We propose that there are distinct seasonal patterns of cardiac mortality and morbidity in south-eastern Australia, and that these display local differences. Consequently, an assessment of seasonal variation, age, and gender differences within the AMI population and where these people live is required if 'high risk' groups are to be identified.

Spatial analysis is often used to describe patterns of infectious disease, cancer and air pollution epidemiology [23-27]. It has been applied less often to analysis of cardiac disease [28,29] and seasonal patterns of coronary mortality and morbidity have not been described from a spatial perspective.

Our aim was to establish the existence of a seasonal pattern in AMI morbidity in Melbourne. In addition, we describe the demographic groups associated with AMI morbidity in the Melbourne region, and represent this information spatially.

A strength of the present study was that it was comprehensive for the population studied, as it included all AMI admissions to 37 hospitals in the Melbourne metropolitan area.

## Methods

### Measures

The morbidity measure used in this analysis was the daily number of hospital admissions for AMI in Melbourne Australia. Melbourne is Australia's second largest city. The CBD and suburbs comprise the Statistical District (SD) of Melbourne with an estimated resident population of 3,488,750 persons [30]. The study population consisted of all subjects hospitalised in the 37 hospitals in the SD of Melbourne during a 6-year period from 1/1/1999–31/12/2004 who were aged 35 years and older, were resident in the SD of Melbourne, and had a principal diagnosis of AMI.

The International Classification of Disease version 10 codes (I21.0, I21.1, I21.2, I21.3 I21.4, and I21.9) were used to define AMI. The ICD-10 classification for I21.1–9 includes all myocardial infarction specified as acute or within a stated duration of 4 weeks or less from onset. This does not include certain current complications or treatments following acute myocardial infarction, myocardial infarction (old), – specified as chronic or with a stated duration of more than 4 weeks from onset, or subsequent post myocardial infarction syndrome. It is possible that there are cases of readmission relating to the initial AMI soon after the incident. However, our understanding is that all admissions were for AMI and repeat admissions would be excluded by different coding for each subsequent admission for investigation and or treatment.

Data were supplied by the Department of Human Services (DHS) from the Victorian Admitted Episode Dataset (VAED) and included all five Melbourne hospital regions, age (in 5-year groups), sex, date of admission to hospital, principal diagnosis, and the Statistical Local Area in which each patient resided. The VAED is audited internally every two years and has a published 0.9% error rate [30]. Definition of the age distribution was determined by generating a frequency distribution using SPSS [31] to assess the percentage contribution of each age group to the entire AMI cohort. Ninety-nine percent of the AMI admissions occurred in persons aged 35 years and older; this group was taken as representative of the AMI cohort.

To accommodate for population increases over time, daily and monthly rates of AMI admissions were calculated using estimated mid-year residential population (ERP) for each cohort per 100,000 persons [32]. A Monthly AMI ratio of mean monthly observed AMI admissions and expected long-term mean monthly admissions was generated to demonstrate changes in trend and seasonality that may be related to changes in diagnosis.

Patient characteristics examined were sex and age. Four groups were examined initially, younger males 35–54 years, older males 55 years and older, younger females 34–54 years, and older females 55 years and older. Each gender grouping was then divided into four age categories 35–54 years, 55–64 years, 65–74 years, and 75 years and older. Age-standardised rates were calculated for each group for each year of the study. All statistical analysis was carried out using SPSS version 14.0.1. Statistical Package for Social Science [31]. To examine the joint effects of age group, sex, and month on AMI admissions a general linear model (GLM) was completed [31]. Single sample *t*-tests were used to compare monthly means relative to base month December. The mean monthly admission rate per 100,000 persons was calculated for each month of the 6-year period (i.e. mean January admission rate is the mean of the daily admission rate for all Januarys 1999–2004). Months with a statistically significant increase in AMI admissions when compared with the base month (month with the lowest AMI rate) are referred to as the maximal AMI season, the remaining months are referred to as the minimal AMI season. The percentage difference in mean monthly admissions rate between the base month (month with the lowest admission rate) and all other months was calculated using Excel version 11 [33].

### Weather

Mean monthly temperature data for the Melbourne Regional Office were obtained from the Bureau of Meteorology website [34]. The mean monthly temperatures are derived from long-term records for this site from 1855–2003.

### Spatial analysis

Maps were generated using GeoDa [35], and age specific rates were calculated using estimated mid-year residential population in 5-year age groups for each statistical local area (SLA). The geographic scale of the analysis had the potential to influence the results, therefore a Standardised Incidence Ratio (SIR) was calculated for each SLA in Melbourne (75 in total) using the indirect method to account for the uncertainty about the stability of age-specific rates in areas with small populations (the indirect method of age standardisation is a comparison of the number of observed cases compared with the number of expected cases if the age-specific incidence rates of the standard population are applied to the study population) [36]. This calculation was completed for the entire study period and for both the minimal and maximal AMI seasons independently. Estimated mid-year resident population for the SD of Melbourne was used as a reference population. Exploratory spatial data analysis (ESDA) methods [37] were used to study the spatial patterns of AMI admissions during both the minimal and maximal AMI admissions seasons. This involved using a univariate Moran's I statistic[37]. The variable of interest was entered as SIR in each SLA for both the minimal and maximal AMI seasons. The Moran's I statistic is a measure of spatial autocorrelation for variables with both ratio and interval scales. This statistic is similar in interpretation to the Pearson's Product Moment correlation statistic for independent samples, in that both statistics range between -1.0 and 1.0 depending on the extent and direction of the correlation. The global measure of Moran's I is defined as:

$$I=\sum_{i}\sum_{j}W_{ij}(X_{i}-\mu )(X_{j}-\mu )/\sum_{j}{\left(X_{j}-\mu \right)}^{2}$$

Where *W*_*ij *_is the row-standardised contiguity matrix, *X*_*i *_is the risk scale measure at SLA I, and *X*_*j *_is the risk scale measure at SLA*j*, and *μ *is the average level of risk.

Global Moran's I statistic indicates the level of spatial autocorrelation or overall clustering within the entire geographical area by calculating a pseudo-p value. These are the result of complex permutation methods to determine significant differences between spatial units [37]. This analysis used 9999 permutations for each Moran significance or cluster map. Univariate global Moran's I uses only one variable in the analysis for example SIR and the spatially weighted value of SIR, bivariate global Moran's I determines the strength and direction of the relationship between two variables such as SIR and the age distribution of the population in each SLA.

Local spatial measures of association (LISA) provide information relating to the location of spatial clusters and the types of spatial correlation. Statistics focused on the local level are important because the magnitude of spatial autocorrelation is not necessarily uniform over the study area [38]. The local measure of Moran's I is defined as:

$$I=\frac{\left(X_{i}-\mu \right)}{\sum {\left(X_{i}-\mu \right)}^{2}}\sum_{j}W_{ij}(X_{j}-\mu )$$

As discussed by Anselin (1995, 2006) and Sridharan (2007) this involved two steps;

1. Visualisations of the spatial distribution of AMI SIR for both the minimal and maximal seasons. Spatial units used were SLA. The spatial size of SLA across Melbourne varies considerably.

2. ESDA was completed in GeoDa using the Moran's I statistic, focusing on both the global and local relationships between AMI rates per SLA for minimal and maximal seasons.

a. The Global Moran's I statistic was used to measure the overall clustering.

b. The Local Moran's I statistic (local indicators of spatial association LISA) indicated the areas of local clusters and spatial outliers. These are presented as significance maps and/or cluster maps in this analysis.

c. Significance was tested by comparison to a reference distribution obtained by random permutations (9999 times).

d. Tests for positive/negative spatial autocorrelation were made.

e. Spatial contiguity was assessed as Rooks or Queens contiguity [39] (Rooks Weight matrix defines spatial neighbours as those areas with shared borders; for data on a regular grid this would mean cells to the north, south, east, and west of each cell. Queens Weight matrix – defines spatial neighbours as those areas with shared borders and vertexes. For data on a regular grid this would include cells to the north, northeast, east, southeast, south, southwest, west, and northwest) [39].

The extent to which the spatial distribution of AMI admissions is affected by the age structure of the population was assessed using a Bivariate Moran's I and LISA. The variables included were SIR (maximal, minimal seasons) and the percentage of the population aged over 55 years for each SLA. The Bivariate Moran's I is represented as the values of the SIR averaged across all neighbouring locations and plotted against the percentage of population over 55 years in each SLA. If the slope on the scatter plot is significantly different to zero then there is a relationship between SIR and the age structure of the population [27]. The Moran's significance and cluster maps incorporate information from the Moran scatter plot about the significance level of the spatial relationships.

Autocorrelation on all Moran's I scatter plots is divided into four types; these are represented spatially on Moran's cluster maps:

**High – High **High value of SIR in SLA where neighbouring SIRs are also High.

**Low – High **Low value of SIR in SLA where neighbouring SIRs are High.

**Low – Low **Low value of SIR in SLA where neighbouring SIRs are also Low.

**High – Low **High value of SIR in SLA where neighbouring SIRs are Low.

## Results

Age and gender distribution in the AMI cohort are summarised in Table 1. Whilst male AMI admissions are distributed relatively equally across all age groups, female admissions are considerably older with 54.7% of admissions recorded as 75 years or older. There were 33,165 admissions over 2186 consecutive days.

**Table 1:** Total number and (%) AMI admissions to hospital in Melbourne

Sex	Total	35–54 years	55–64 years	65–74 years	75 years and older	55 years and older
Male	21634	5421 (25.1)	5102 (23.6)	5358 (24.8)	5765 (26.4)	16216 (75)
Female	11530	1089 (9.5)	1433 (12.4)	2696 (23.5)	6302 (54.7)	10431(90.4)
Total	33165	6510 (19.6)	6535 (19.7)	8054 (24.3)	12065(36.3)	26653(80.3)

The sex ratio of the AMI cohort varies markedly from that of the Melbourne population, especially with respect to the four age subgroups. Admissions are 2 – 5 times more likely to be male. This does not replicate the sex ratio in the entire Melbourne population, which is approximately equal to one in all age groups except the oldest group where there are approximately 40% more females. This sex imbalance is reflected in the age 75 years and older group when admissions are marginally more likely to be female (0.9: 1).

**Figure 1:**
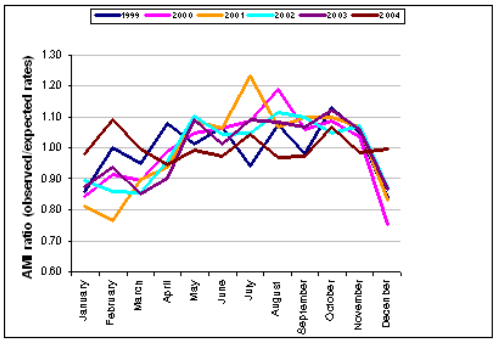
Monthly AMI admissions ratio for each year 1999–2004.

Changes in AMI diagnosis due to the introduction of new diagnostic technology during the study period may have influenced the trend and seasonal pattern. Figure 1 presents monthly patterns in AMI admissions for each year of the study period. As suggested there does not appear to a seasonal effect associated with diagnostic changes.

**Figure 2:**
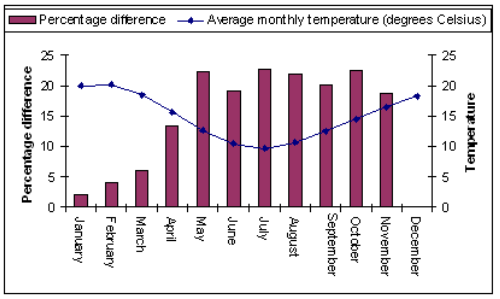
**Monthly percentage difference for AMI admissions rates between each calendar month and base month December and average monthly temperature**.

A comparison of the percentage difference in monthly AMI admissions with the long-term mean monthly maximum and minimum temperature from the Melbourne Regional weather station demonstrates an inverse relationship between mean monthly AMI admissions and mean monthly temperature with an increase in AMI admission during the colder months of the year (see Figure 2).

The monthly percentage difference between the base month and each calendar month across the year for persons aged 35 years and older clearly demonstrates a seasonal pattern with a statistically significant increase (*t *= 3.6 – 7.6, p < 0.001) in the admission rate during April to November. The peak month is July with a 22.7% increase over the base month of December. This pattern of increased admission rates April to November is broader than the traditional winter months of June to August. The period April to November was referred to as the maximal AMI season and months December to March are referred to as the minimal AMI season.

**Figure 3:**
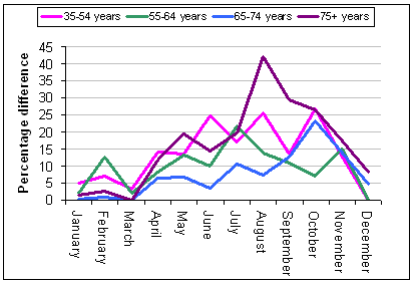
Percentage difference between peak and base months showing the seasonal patterns in AMI admissions for males in each of the four age groups.

**Figure 4:**
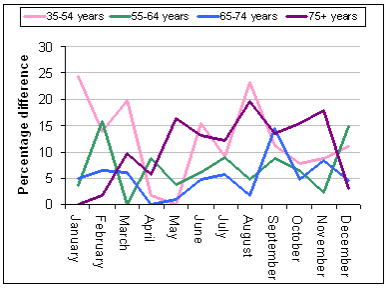
**Percentage difference between peak and base months showing the seasonal patterns in AMI admissions for females in each of the four age groups**.

The seasonal patterns for each sex and age sub-group are shown in Figures 3 and 4. The patterns are most pronounced for the oldest age group 75 years and older. Males demonstrate a more consistent seasonal pattern across all age groups, whereas only females 75 years and older show a clear seasonal pattern.

### Males

**Figure 5:**
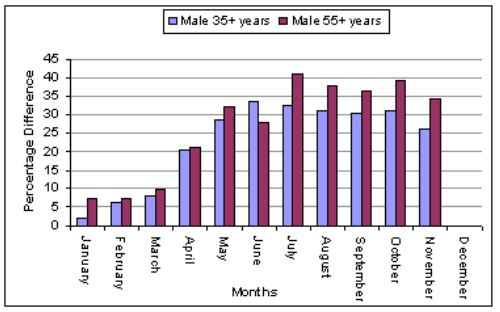
Percentage difference between each calendar month and the base month December for male AMI admissions aged 35 years and older and 55 years and older.

The analysis of male admissions indicated that the two largest male age groups, defined as 35 years and older and 55 years and older, show a broad seasonal pattern with increased rates during the cooler months with the months of peak occurrence skewed towards the spring for males over 55 years of age as shown in Figure 5. However, age sub-groupings within this group display some variation in the seasonal response (details are shown in Table 2 and Figure 3).

**Table 2:** Percentage difference from peak to base months across the year for male and female AMI admissions in four age groups

Sex	Age groups			
	
	35–54 years	55–64 years	65–74 years	75+ years
Male	26.8%	20.8%	24.1%	42.6%
Female	24.3%	15.3%	14.7%	22.3%

For males 35 years and older the maximal AMI season extends from April to November with a percentage difference across the year of 33.7%. The peak occurrence is in June (*t *= 7.88, p < 0.001) with a mean monthly admission rate of 0.95, (SD = 0.04). Males 35–54 years demonstrate an increase in AMI admissions during the colder months; the pattern is irregular but extends from April to November. The peak month is October with a 26.8% increase in admissions over the base month March. For males older than 55 years the maximal AMI season extends from April to November (*t *= 7.13 – 3.24, p = < 0.001). The peak month is July with a mean admission rate of 2.43, (SD 0.95). The 55 years and older cohort was divided into three age groups. The seasonal difference in AMI admissions is most pronounced in the oldest group (see Table 2 and Figure 3) with a 42.6% change in mean monthly admissions rate across the year.

### Females

Seasonal analysis of female age groups also demonstrated a seasonal response for the two main age categories of 35 years and older and 55 years and older, with some differences between the age sub-groups. Females show a sharp increase in AMI admissions in May and a seasonal increase that extends from May to November (*t = *5.03, p < 0.001) with a percentage difference of 23.1% across the year. Females aged less than 75 years do not show a consistent seasonal increase in AMI admissions across the year see Figure 4. Females 75 years and older demonstrate a strong seasonal pattern from May to November with a 22.3% difference between peak and base months as shown in Table 2 and Figure 4. This group comprises 60% of the female admissions and appears to be driving the seasonal pattern observed for females 35 years and older.

Females 35–54 years demonstrate a large difference between peak and base months 24.3%. However, there is no apparent seasonal pattern in this group. This may be due to low numbers of admissions for this group (n = 1089, or 9.6% of female cohort). Peak admissions occur during the warmer months January to March and again in August.

**Figure 6:**
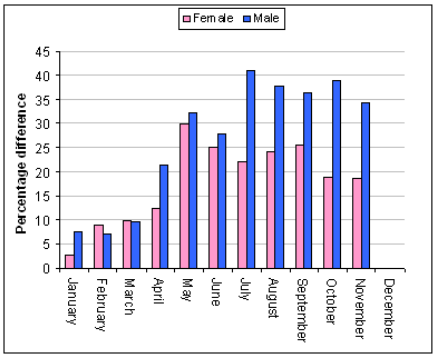
Percentage difference in monthly AMI admissions for males and females aged 55 years and older 1999–2004.

The gender differentiation observed between monthly male and female admissions for persons aged 55 years and older shown in Figure 6 demonstrated a significant difference (*t *= -18.8, df = 11, p < 0.001). Both groups show increased admissions rates during the colder months, but males demonstrate a greater percentage difference during the colder months and during spring. The seasonal peak in AMI admissions as shown in Figure 6 was also different as male admissions peaked in July (41% difference between peak and base months) and female admissions peaked in May (29.9% difference between peak and base months).

### Spatial visualisation

**Figure 7:**
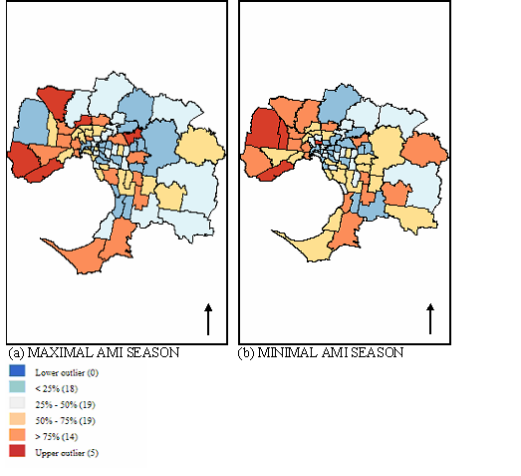
AMI admissions during (a) maximal and (b) minimal AMI seasons in Melbourne during study period 1999–2004 (number of SLAs in parenthesis).

Maps shown in Figure 7 demonstrate the spatial differences in the SIR of the seasonal distribution of AMI admissions in Melbourne. These maps display the spread or distribution as interquartile ranges of the SIR across Melbourne. SIRs are grouped into four categories plus high and low outliers; values are categorised as outliers if they are 1.5 times the value of the interquartile range.

**Figure 8:**
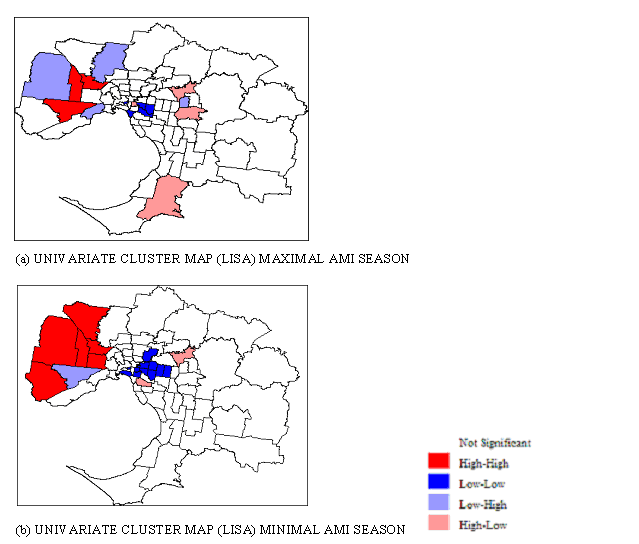
**Univariate Moran's I (LISA) cluster maps for SIR of AMI admissions in (a) maximal and (b) minimal season**.

The map in Figure 7(b) demonstrates the spatial concentration of SIR in the western and north-western suburbs during the minimal AMI season. The null hypothesis (spatial distribution was random) was tested for spatial autocorrelation using a Global Moran's I. The results are summarised in Table 3, with significance values based on the permutation approach (9999 permutations). The results suggest that there is a degree of spatial patterning during both the maximal and minimal AMI seasons. However, the spatial clustering during the minimal season is more pronounced. Further analysis using LISA contributed to understanding the spatial relationships for both seasons. The cluster spatial typology (high-high, low-low, etc) outlined earlier was completed for both AMI seasons. Cluster maps based on LISA are shown in Figure 8 and reveal six areas with high-high typology in the western and north-western regions of Melbourne during the minimal season (Figure 8(b) and three SLA of high-high typology in the same region during the maximal AMI season Figure 8(a).

**Table 3:** Moran's I statistic for the Global spatial autocorrelation of SIR (largest pseudo p value)

Spatial weight	Maximal	Minimal
**Univariate**		

Queens contiguity	0.0455 (0.01)	0.3468 (0.0001)
Rooks contiguity	0.0453 (0.01)	0.3463 (0.0001)

Spatial weight	Maximal	Minimal

**Bivariate **(SIR*Age)		

Queens contiguity	-.1432 (0.01)	-.2248 (0.01)
Rooks contiguity	-.1428 (0.01)	-.2242 (0.01)

**Figure 9:**
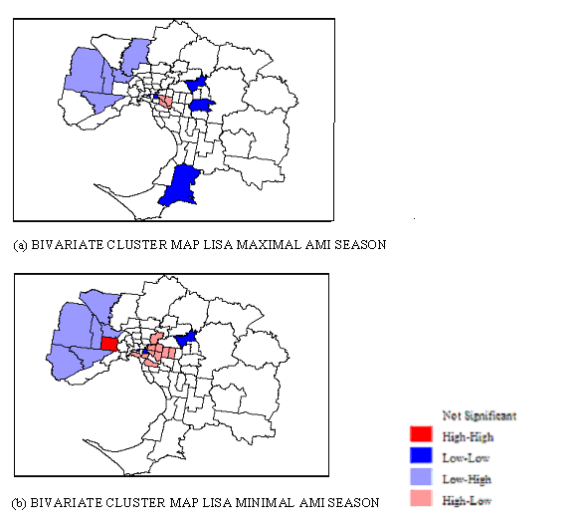
**Bivariate Moran's I (LISA) cluster maps for SIR of AMI admissions and age during the (a) maximal and (b) minimal seasons**.

The Bivariate spatial relationship between age SIR was studied using the Bivariate Moran's I [27]. The Bivariate Moran's I represents the value of the age variable (percentage of the population aged over 55 years) averaged across neighbouring locations and plotted on the variable for SIR. If the slope on the scatter plot is significantly different from zero then there is a bivariate spatial relationship between age and SIR. The contribution of the age structure of the population in each SLA to the spatial patterns of AMI admissions described is shown in Figure 9. Both maps indicate that areas with more than expected AMI admissions have lower numbers of people over the age of 55 years. The Bivariate Moran's I statistic for minimal and maximal seasons indicates a weaker relationship between SIR and age during the minimal season (see Table 2); that is during the minimal season, areas with increased SIR have a lower percentage of persons aged 55 years and older. However, during the minimal season one area (Brimbank-Sunshine) demonstrates high numbers of people over 55 years of age and high SIR. By contrast, the areas in inner eastern Melbourne with high total numbers of people over 55 years have low SIR.

## Discussion

The main aim of this paper was to describe the seasonal, demographic, and spatial patterns of AMI admissions to hospital in Melbourne between 1999 and 2004. This is the first study to demonstrate demographic and spatial differences in the seasonal patterns in AMI admissions to hospital in Melbourne.

Male and female admissions demonstrate a classical seasonal response with higher rates during the colder months particularly leading into spring, and lower rates during the warmer months. The mean monthly admission rates were lower in the female group than in the corresponding male group; in addition, the seasonal pattern was less pronounced for females with the percentage difference between peak – base month of 29% whereas the corresponding difference for males was 41%.

Studies from the northern hemisphere have indicated that winter mortality is greater for women rather than men [1]. The Eurowinter Group suggests that this may be due to women wearing less suitable clothing such as skirts during cold weather [40]. The reason for this reversal is not clear but may be related to cultural factors specific to the Melbourne population or that this study only addresses AMI admissions not cardiac mortality or mortality in general.

For people aged 55 years and older, the peak AMI admission month is May in older females but in older males the peak month is July. The age*sex analysis of the 55 years older group again demonstrates differences in age distribution, and seasonal distribution. Whereas 90% of female admissions are in the older than 55 years group, only 75% of male admissions are in this category.

In this study, male AMI rates demonstrate the most pronounced seasonal distribution. This contrasts with reports from the northern hemisphere where females show a stronger seasonal pattern than males especially in areas with milder winters [1,14]. Increased AMI admissions rates were also noted for both males and females in the 55–64 years age group during February, indicating a possible relationship between hot weather and AMI admissions in an ageing but not elderly population. Many people in this age group may still be employed and this seasonal pattern might represent environmental/occupational exposure to heat. There were not heat waves recorded in Melbourne during this period.

A seasonal increased admissions rate from May to November is evident in males older than 55 years and females older than 65–74 years; this is skewed towards the spring months of September, October, and November. Winter increases in respiratory disease and biomedical risk factors [41-48] related to decreasing temperature would be the primary explanation for this pattern. However, the increases noted here are typically in spring after the coldest part of the year has passed. A possible explanation may be that many retired Melbournians holiday in the northern states during the winter period and we speculate that this spring peak may be related to their return to Melbourne. After several months holiday in a warm climate the abrupt change to a cooler climate and returning home to an unkempt garden and home may incur physical exertion enough to trigger an MI. There is certainly enough evidence provided by these analyses to investigate this phenomenon further. It would also be interesting to investigate the admission rates of AMI in southeast Queensland and the mid-north coast of New South Wales during the winter months, noting Melbourne SLA or postcodes as the place of residence recorded in admitted episodes datasets.

The percentage of male AMI admissions in each of the three age groups in the older than 55 years group is evenly distributed, unlike the corresponding female group where 60% of admissions are aged 75 years or older. The seasonal distribution in the overall female admissions appears to be influenced by the strong seasonal pattern in this older group. This seasonal pattern amongst the elderly may be due to cold exposure either inside or outside the home, or associated illness i.e. influenza or increased susceptibility to both cold and infectious diseases due to co-morbidities such as diabetes and respiratory disease.

With regard to the younger age groups, the pattern of mean monthly admission rates during the maximal season is erratic for both males and females. This may be a result of the dynamics within the 'susceptible pool' and the pool size itself. The pool may be quite small but the rate at which people enter and leave the pool may be quite high. Females 35–54 years compose 9.5% of the female cohort and this group demonstrated an irregular pattern throughout the year, not a consistent seasonal pattern. However, they do display a 24% difference between peak (January) and base (May) months with increased admission during the warmer months. This group of patients is rarely described as studies predominantly concentrate on older groups [15,49-54].

The rates in the youngest age group are of concern with an average of 2.7 (male) and 0.5 (female) hospital admissions for AMI per day. This suggests an increase in the socio-economic burden of disease and increased DALYS (disability adjusted life years) in this group. The spring peak in October in males and the summer increase in younger female admissions also suggest that the trigger may be more complex than cold winter weather and suggests that associated co-morbidity, air pollution, behavioural changes, and/or increased environmental exposures in susceptible persons may also be important factors in determining seasonality.

In short, there are marked differences in the seasonal distribution of AMI admission by both gender and age. With the exception of female admissions less than 75 years, the seasonal distribution of AMI admissions increases during the colder months of late autumn, winter, and spring. The seasonal pattern is much broader in Melbourne than that described in northern hemisphere reports [10,55]. Seasonal differences are often described for the 3–4 month winter period in the northern hemisphere. Extended seasonal patterns in Australia have also been reported for New South Wales [20,56]. However, in Melbourne the seasonal increase extends for up to eight months. This suggests that the observed response is more complex than initially thought and responsive to triggers of AMI other than temperature alone.

The impacts of possible external triggers of AMI are described by Culic et.al (2005) AMI events can be triggered by physical activity, emotional stress, and sexual activity, and excessive eating [57]. However, the frequency and intensity of these events preceding AMI varies between many of the published reports. Further insight into the relationships between external triggers and pathophysiologic mechanisms of AMI onset could help develop preventative strategies to reduce its incidence.

The results presented here are consistent with a growing body of knowledge that highlights the effects of space and place on population health. Despite these findings, epidemiological research has paid little attention to the effects of place-based characteristics on population health [23]. Therefore, a great deal more is known about the population demography of health rather than the spatial characteristics of health, or the effect the latter has on exposure. A matter of interest in this study was the spatial patterning of seasonal AMI admissions to hospital, and the suggestion that AMI admissions may not be drawn from the same population throughout the year. This would suggest that variables other than temperature alone might be implicated in the observed seasonal distribution of AMI admissions. The spatial clustering observed in the northern and western suburbs of Melbourne is more pronounced during the warmer months of the minimal season as demonstrated using LISA. The mechanisms responsible for this phenomenon are difficult to determine at this broad level of analysis. However, there are almost certainly a series of social and environmental processes, which involve the combined aspects of neighbouring areas, and contribute to the observed spatial clusters. For example, the north-western suburbs of Melbourne are built on an exposed basalt plain; this is an industrial region and has experienced increased urbanisation during the recent past, with low cost housing.

Analysis of the age distribution in Melbourne SLA and AMI admissions during both seasons indicated that although AMI admissions rates increased with age, advancing age did not explain the spatial distribution of admissions, as the areas with the highest percentage of aged persons did not have the highest SIR. Therefore, the observed patterns are likely to be representative of socioeconomic status and the distribution of cardiac disease in conjunction with features of the built environment and the geography of the northern and western suburbs, such as flat basalt plains, industrialised regions and urban heat island effects.

## Concluding comments

This study has some limitations; it only includes admissions to hospitals and to aged care facilities and did not include out-of-hospital deaths. Diagnoses were based on the ICD-10 classification system and coding by medical clerks from clinician records. In addition, the study could not analyse AMI mortality or AMI outcomes following hospitalisation. Some analyses, particularly for the younger female population, may have lacked sufficient power to detect seasonal patterns due to small numbers of admissions.

The results presented indicate that there is a classical seasonal pattern with increased admissions during the colder part of the year. Persons admitted to hospital with AMI tend to be older and males present a stronger seasonal pattern than females. Whist some areas to the north and west of Melbourne have high rates of AMI admissions during both the warmer and colder months there are significant differences in the spatial distribution of AMI admissions throughout the year that cannot be explained by the age structure of the resident population. The spatial distribution of AMI admissions during the year do demonstrate some differences and this warrants further investigation including; detailing the incidence of cardiac disease in the community and the consideration of socioeconomic circumstance.

## Competing interests

The authors declare that they have no competing interests.

## Authors' contributions

MEL contributed to the initial study design, data acquisition, and analysis, interpretation of results and drafting of the maps and the manuscript. NN provided advice relating to analysis of data and interpretation of the results and helped draft the manuscript. NT contributed to the study design, analysis and interpretation of results and helped draft the manuscript. All authors have read and approved the final manuscript.
